# New insights on the interplays between m^6^A modifications and microRNA or lncRNA in gastrointestinal cancers

**DOI:** 10.3389/fcell.2023.1157797

**Published:** 2023-06-19

**Authors:** Tao Su, Jiandong Liu, Nasha Zhang, Teng Wang, Linyu Han, Suzhen Wang, Ming Yang

**Affiliations:** ^1^ Medical Integration and Practice Center, Cheeloo College of Medicine, Shandong University, Jinan, Shandong, China; ^2^ Shandong University Cancer Center, Jinan, Shandong, China; ^3^ Shandong Provincial Key Laboratory of Radiation Oncology, Cancer Research Center, Shandong Cancer Hospital and Institute, Jinan, Shandong, China; ^4^ Department of Radiation Oncology, Shandong Cancer Hospital and Institute, Shandong First Medical University and Shandong Academy of Medical Sciences, Jinan, Shandong, China; ^5^ Jiangsu Key Lab of Cancer Biomarkers, Prevention and Treatment, Collaborative Innovation Center for Cancer Personalized Medicine, Nanjing Medical University, Nanjing, Jiangsu, China

**Keywords:** m^6^A modification, RNA modification, ncRNA, microRNA, lncRNA

## Abstract

N^6^-Methyladenosine (m^6^A) methylation is one of the most extremely examined RNA modifications. M^6^A modification evidently impacts cancer development by effecting RNA metabolism. Long non-coding RNAs (lncRNAs) and microRNAs (miRNAs) are involved in multiple essential biological processes by regulating gene expression at the transcriptional and post-transcriptional levels. Accumulated evidences indicated that m^6^A is involved in regulating the cleavage, stability, structure, transcription, and transport of lncRNAs or miRNAs. Additionally, ncRNAs also play significant roles in modulating m^6^A levels of malignant cells by participating in the regulation of m^6^A methyltransferases, the m^6^A demethylases and the m^6^A binding proteins. In this review, we systematically summarize the new insight on the interactions between m^6^A and lncRNAs or miRNAs, as well as their impacts on gastrointestinal cancer progression. Although there are still extensive studies on genome-wide screening of crucial lncRNAs or miRNAs involved in regulating m^6^A levels of mRNAs and disclosing differences on mechanisms of regulating m^6^A modification of lncRNAs, miRNAs or mRNAs in cancer cells, we believe that targeting m^6^A-related lncRNAs and miRNAs may provide novel options for gastrointestinal cancer treatments.

## Introduction

RNA modifications play pivotal roles in regulating stability and functions of various RNAs. RNA methylation is one of the most extensively and diligently studied RNA modifications. Previous reports have shown that N6-methyladenosine (m^6^A) modification affects tumor development and progression in a variety of ways, such as influencing cell proliferation, promoting of cancer stem cell regeneration, and leading to resistance of malignant cells to radiotherapy or chemotherapy (Lan et al., 2019a). Another abundant mRNA modification is N6,2-O-dimethyladenosine (m^6^A.m.), which occurs near the mRNA cap (Wei et al., 1975). M^6^A.m. negatively impacts cap-dependent translation of methylated mRNAs (Sendinc et al., 2019). In addition, m^6^A.m. has been identified as an internal modification and a crucial regulatory factor in snoRNA (Chen et al., 2020; Goh et al., 2020; Oerum et al., 2021). However, m^6^A is the main RNA modification in long non-coding RNAs (lncRNAs) and microRNAs (miRNAs) (Oerum et al., 2021). In this review, we mainly summarize the correlation between m^6^A modification and lncRNA/miRNA in gastrointestinal cancers.

M^6^A modification is a reversible process, which requires the participation of the m^6^A methyltransferases (writers), the m^6^A demethylases (erasers) and the m^6^A binding proteins (readers) (Fu et al., 2014). The main functions of the m^6^A methyltransferases, such as WTAP, METTL3, and METTL14, are to initiate methylation of adenosines in RNAs. The m^6^A demethylases, such as FTO and ALKBH5, are to recognize and delete m^6^A modification. The m^6^A binding proteins including YTHDC1/2, YTHDF1/2/3 and IGF2BP1/2/3, can recognize methylated base pairs and impact cellular functions including mRNA translation, RNA metabolism, as well as interactions between microRNA (miRNAs) and their target RNAs (Nettersheim et al., 2019). Divergent cell-type-specific and tissue-specific expression and localization of these proteins results in significant differences in the level of m^6^A modification in different cell types (Shi et al., 2019). In addition, environmental factors also play important regulatory roles in this process (Shi et al., 2019).

M^6^A modification is considered to be a critical posttranscriptional regulator of gene expression. M^6^A modifications of messenger RNA (mRNA) regulate various aspects of mRNA metabolism (Frye et al., 2018). Non-coding RNAs (ncRNAs), such as miRNAs and lncRNAs, have crucial biological functions in the tumorigenesis and progression of various types of cancers (Jacob et al., 2017). It has been found that m^6^A modifications on miRNAs and lncRNAs can also regulate their production, metabolism, and functions (Han et al., 2019). Interestingly, miRNAs and lncRNAs were involved in regulating m^6^A modifications as well (Cai et al., 2018). In several pre-clinical studies, targeting m^6^A and miRNAs or lncRNAs in cancer cells simultaneously showed synergistic effects (Wahlestedt, 2013; Zhang et al., 2016). In addition, m^6^A modification can regulate chromatin state via lncRNAs (Wei and He, 2021). For example, in mouse embryonic stem cells (mESCs), FTO mediates m^6^A demethylation of LINE1 regulating LINE1 RNA abundance and the local chromatin accessibility (Wei et al., 2022). In this review, we systematically summarized the interplays between m^6^A modification and miRNAs/lncRNAs, and described the significance how they impact tumorigenesis in gastrointestinal cancers.

## Impacts of m^6^A modifications on lncRNAs

**FIGURE 1:**
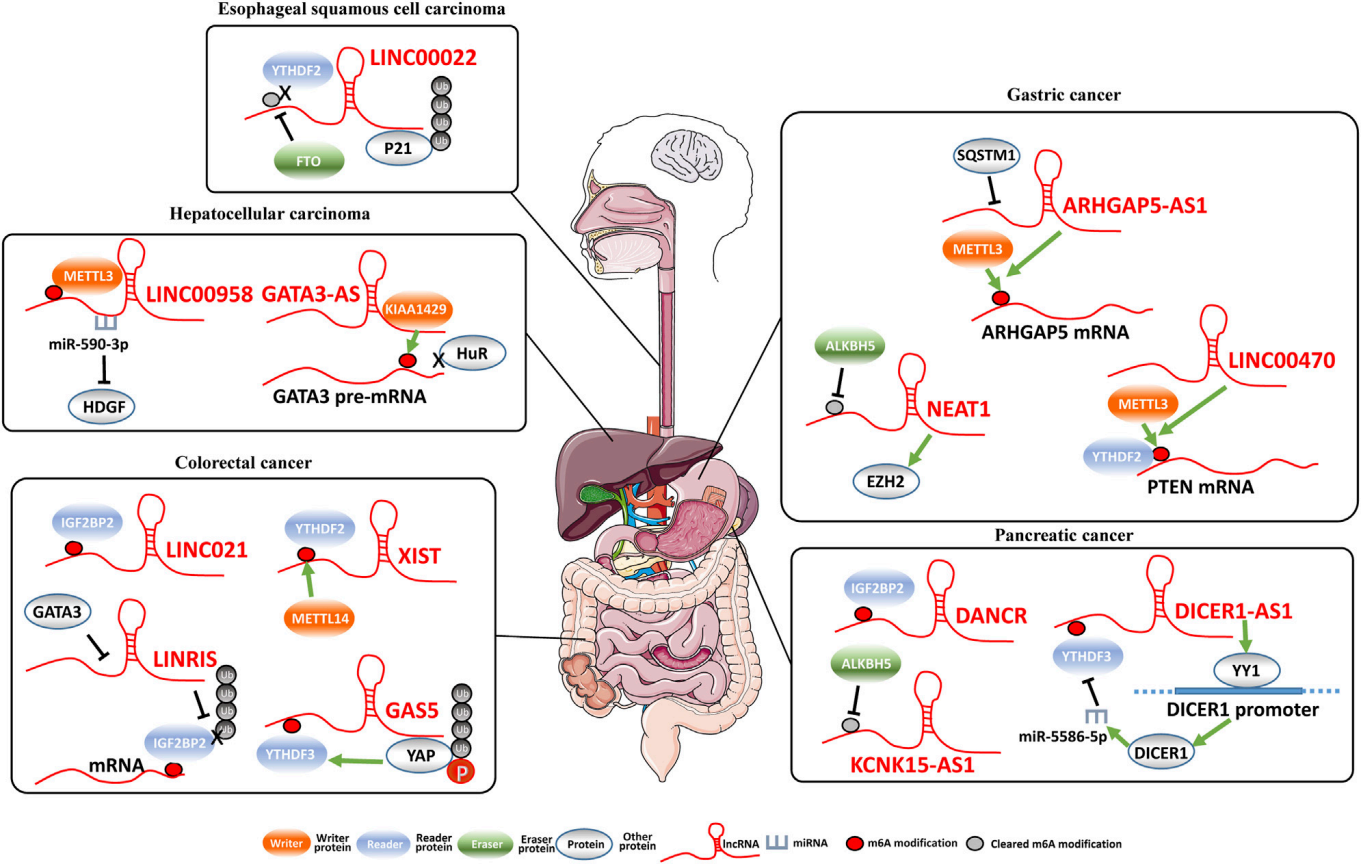
Regulation of lncRNAs by m^6^A modification and lncRNA-regulated m^6^A modification..

LncRNAs are a class of ncRNA molecules with more than 200 nucleotides (nt). Compared with other types of ncRNAs, lncRNAs have more family numbers and more complicated functions (Schmitt and Chang, 2016). In different gastrointestinal cancers, lncRNAs regulate expression of protein-coding genes via distinct mechanisms (Figure 1). There are plenty of m^6^A modifications in lncRNAs, of which show temporal and spatial specificities (Xiao et al., 2019). M^6^A modification can regulate expression and functions of lncRNAs. Proteins involved in m^6^A modifications can impact m^6^A levels of lncRNAs and stability of lncRNAs, which eventually regulates various biological functions of malignant cells. Moreover, m^6^A modification can initiate RNA-protein binding via providing binding sites for m^6^A reader protein or adjusting RNA local structures (Quinn and Chang, 2016), which might also impact lncRNA functions (Table 1).

**TABLE 1:** M^6^A modification regulates lncRNA stability.

Proteins	Protein types	Functions	Target lncRNAs	Tissues	Functional classification	References
FTO	Eraser	Decreased m^6^A levels	LINC00022	ESCC	Promoting tumor growth of ESCC	Cui et al. (2021)
ALKBH5	Eraser	Decreased m^6^A levels	NEAT1	GC	Promoting GC cell proliferation and migration	Zhang et al. (2019b)
METTL3	Writer	Increased m^6^A levels	LINC00958	HCC	Promoting HCC cell proliferation and migration	Zuo et al. (2020)
IGF2BP	Reader	Identify the m^6^A site	DANCR	PC	Promoting cell proliferation and stem cell-like properties	Hu et al. (2020)
YTHDF3	Reader	Identify the m^6^A site	DICER1-AS1	PC	Suppressing glycolysis, proliferation, and metastasis of PC cells	Hu et al. (2022)
ALKBH5	Eraser	Decreased m^6^A levels	KCNK15-AS1	PC	Acting as a tumor suppressor and inhibits malignant behaviors of PC cells	He et al. (2018) He et al. (2021)
METTL14	Writer	Increased m^6^A levels	XIST	CRC	Promoting CRC cell proliferation and migration	Yang et al. (2020)
YTHDF3	Reader	Identify the m^6^A site	GAS5	CRC	Inhibiting cell proliferation, invasion, migration, EMT, and radiation resistance	Ni et al. (2019)

ESCC, esophageal squamous cell carcinoma; GC, gastric cancer; HCC, hepatocellular carcinoma; PC, pancreatic cancer; CRC, colorectal cancer.

### Esophageal squamous cell carcinoma

Esophageal squamous cell carcinoma (ESCC) is one major pathological subtype of esophageal cancer and one of the most lethal cancers worldwide. In East Asia including China, ESCC accounts for more than 90% of all esophageal cancer patients. However, how m^6^A impacts lncRNA functions in ESCC remains largely unexplored. Cui et al. (2021) found that LINC00022, which is demethylated by the m^6^A eraser FTO, promotes tumor growth of ESCC. LINC00022 is upregulated in primary ESCC samples and associated with poor outcomes for ESCC patients. In ESCC cells, LINC00022 interacts with P21 protein and enhances ubiquitination-mediated degradation of P21. The m^6^A eraser FTO reduces m^6^A methylation levels of LINC00022 and results in inhibited LINC00022 decay via the m^6^A reader YTHDF2. Therefore, over-expressed FTO drives LINC00022-dependent ESCC cell proliferation *in vitro* and *in vivo*.

### Gastric cancer

As one of the most common digestive system cancers, gastric cancer (GC) is the leading cause of cancer-related death in the world. LncRNA NEAT1 is overexpressed in GC tissues compared to normal stomach tissues. Consistently, NEAT1 could significantly promotes invasion and metastasis capabilities of GC cells. Interestingly, lncRNA NEAT1 is a target RNA of the m^6^A demethylase ALKBH5. ALKBH5 eliminated the m^6^A modification of NEAT1 and, thus, influences the expression of EZH2 as well as GC progression (Zhang et al., 2019b).

### Hepatocellular carcinoma

In hepatocellular carcinoma (HCC), METTL3-mediated m^6^A modification stabilizes lncRNA LINC00958 to promote HCC progression. METTL3 increases m^6^A modification levels of LINC00958 and inhibits lncRNA degradation. High levels of LINC00958 promotes lipogenesis in HCC cells. Interestingly, LINC00958 upregulates hepatoma-derived growth factor (HDGF) expression through sponging miR-3619-5p (Zuo et al., 2020). As a result, the interaction between METTL3 and lipogenesis-related lncRNA LINC00958 enhances proliferation and metastasis capability of HCC cells.

### Pancreatic cancer

Pancreatic cancer (PC) is the seventh leading cause of cancer death worldwide, with a 5-year survival rate of approximately 10% of patients (Hu et al., 2020). IGF2BPs family, as m^6^A readers, recognized the consensus GG (m^6^A)C motif of thousands of transcripts with m^6^A modification including lncRNAs (Huang et al., 2018). Among them, the RNA-binding protein IGF2BP2 play important roles in RNA localization, stability, and metabolism as a posttranscriptional regulatory factor. To investigate the clinical importance of IGF2BP2 in PC, Hu et al. (2020) examined IGF2BP2 expression through immunohistochemistry staining in the PC tissue microarrays containing 82 cancerous samples and 54 normal specimens. It has been found that evaluated expression of IGF2BP2 is significantly associated with poor outcomes of PC patients and suppression of IGF2BP2 inhibits cell proliferation. Indeed, IGF2BP2 can interact with lncRNA DANCR and enhances DANCR expression in PC cells. LncRNA DANCR has been reported to markedly increase stemness features of malignant cells to promote tumorigenesis in HCC, osteosarcoma and acute myeloid leukemia (Yuan et al., 2016; Jiang et al., 2017; Bill et al., 2019; Nance et al., 2020). Consistently, the high expression of lncRNA DANCR also accelerates PC cell proliferation and differentiation of cancer stem cells (Hu et al., 2020). LncRNA DANCR is modified with m^6^A in PC. IGF2BP2 serves as the reader for the m^6^A-modified DANCR and stabilizes DANCR RNA.

In PC, DICER1-AS1 is another m^6^A modified lncRNA which plays a pivotal role in glycolysis of PC cells (Hu et al., 2022). Overexpressed lncRNA DICER1-AS1 suppresses glycolysis, proliferation, and metastasis of PC cells via transcriptionally enhancing *DICER1* expression. DICER1 enhances miR-5586-5p maturation and subsequently represses expression of multiple glycolytic genes. The m^6^A reader YTHDF3 recognizes the m^6^A-modified DICER1-AS1 and results in lncRNA DICER1-AS1 degradation in response to glucose depletion. Additionally, it has been found that the m^6^A reader *YTHDF3* is a target gene of miR-5586-5p. Together, these results suggest that there is negative feedback with YTHDF3/DICER1-AS1/DICER1/miR-5586-5p to regulate PC glycolysis and pathogenesis.

In PC cells, KCNK15-AS1 is a target lncRNA of ALKBH5 (He et al., 2018; He et al., 2021). KCNK15-AS1 is significantly downregulated in PC tissues. Consistently, lncRNA KCNK15-AS1 acts as a tumor suppressor and inhibits malignant behaviors of PC cells. The m^6^A eraser ALKBH5, which was downregulated in PC cells, demethylates KCNK15-AS1 and regulates KCNK15-AS1-mediated cell motility (He et al., 2018). Moreover, He et al. (2021) found that lncRNA KCNK15-AS1 could bind with *KCNK15* mRNA 5′-UTR to inhibit *KCNK15* translation. Moreover, lncRNA KCNK15-AS1 recruits the E3 ligase MDM2 to REST protein, promotes REST ubiquitination and, thus, transcriptionally upregulates tumor suppressor PTEN expression to inactivate the AKT signaling (He et al., 2021).

### Colorectal cancer

Colorectal cancer (CRC) is one of the leading cancer types, ranking third for incidence, but second for mortality, and the incidence rate is rising worldwide. In CRC, the reduced levels of METTL14 expression were also markedly correlated with unfavorable prognosis of patients (Yang et al., 2020). Consistently, METTL14 could repress proliferative and invasive capabilities of CRC cells *in vitro* and *in vivo*. LncRNA XIST is a target of METTL14 in CRC. Knockdown of METTL14 substantially decreases oncogenic lncRNA XIST m^6^A modification levels and upregulates XIST expression. YTHDF2 acts as the m^6^A reader protein of XIST and mediates degradation of m^6^A-modifed XIST. In line with this, there was significantly negative expression correlations between lncRNA XIST and METTL14 or YTHDF2 in CRC tissues. These results highlighted the function and prognostic values of m^6^A modification and lncRNA XIST in CRC.

YAP activation is crucial for CRC tumorigenesis and progression (Johnson and Halder, 2014). Ni et al. identified GAS5 as a YAP-interacting lncRNA via analyzing the RIP-seq profiles (Ni et al., 2019). In CRC tissues, the expression levels of lncRNA GAS5 are negatively correlated with YAP. GAS5 directly interacts with the WW domain of YAP, thereby promoting endogenous YAP transport from the nucleus to the cytoplasm. In line with this, lncRNA GAS5 promotes YAP phosphorylation and subsequently ubiquitin-mediated YAP degradation, and thus inhibiting the CRC progression *in vitro* and *in vivo*. As one of the m^6^A “readers”, YTHDF3 collaborates with YTHDF1 to promote translation of protein synthesis and influence decay of methylated mRNA mediated through YTHDF2 (Shi et al., 2017; Ni et al., 2019). Notably, YTHDF3 recognizes the m^6^A modification site of lncRNA GAS5 and promotes its degradation, while GAS5 can repress YAP-mediated YTHDF3 transcriptional expression. In all, the study proposed a negative feedback regulation of lncRNA GAS5-YAP-YTHDF3, and established a new mechanism for m^6^A-induced degradation of lncRNA (Ni et al., 2019).

## LncRNA-regulated m^6^A modifications

As described previously, the m^6^A modification of lncRNAs could impact expression and metabolism of lncRNAs. Interestingly, lncRNAs are also involved in regulation of expression of m^6^A-related proteins as well as functions of m^6^A-modified mRNAs at the post-transcriptional level in various cancers (Table 2; Figure 1).

**TABLE 2:** LncRNA-regulated m^6^A modification.

LncRNAs	Cancers		Proteins	Functions of lncRNAs	Functional classification	References
ARHGAP5-AS1	GC		METTL3 HUR	Recruiting METTLE3 and stimulating m^6^A modification on ARHGAP5 mRNA	Enhancing chemoresistance of GC cells	Zhu et al. (2019)
LINC00470	GC		METTL3 YTHDF2	Increasing m^6^A modification on PETN mRNA decreasing its stability and degrading m^6^A-dependent reader protein YTHDF2	Promoting GC cell proliferation and migration	Yan et al. (2020)
GATA3-AS	HCC		KIAA1429	Acting as a cis-acting element for the interaction of KIAA1429 with GATA3 pre-mRNA.	Promoting the proliferation and cancerigenicity of HCC	Lan et al. (2019b)
LINRIS	CRC		IGF2BP2	Reducing IGF2BP2 ubiquitination	Promoting aerobic glycolysis in CRC	Wang et al. (2019)
LINC021	CRC		IGF2BP2	Binding with the m^6^A reader IGF2BP2 protein and enhanced the mRNA stability of MSX1 and JARID2	Promoting CRC malignant proliferation, migration capabilities, and reduced cell apoptosis	Wu et al. (2022)

GC, gastric cancer; HCC, hepatocellular carcinoma; CRC, colorectal cancer.

### Gastric cancer

LncRNAs could impact the stability of their target RNAs by recruiting m^6^A writer proteins. For instance, lncRNA ARHGAP5-AS1 is highly expressed in chemoresistant GC cells and silencing ARHGAP5-AS1 reversed chemoresistance (Zhu et al., 2019). LncRNA ARHGAP5-AS1 can stimulate transcription of *ARHGAP5* in the nucleus of GC cells, whereas stimulate m^6^A modification of *ARHGAP5* mRNA to stabilize it through recruiting the m^6^A writer METTL3 in the cytoplasm. Interestingly, SQSTM1 can translocate lncRNA ARHGAP5-AS1 to autophagosomes to accelerate its degradation in cells. Therefore, the ARHGAP5-AS1/ARHGAP5 axis might be a possible target to reverse chemoresistance of GC cells.

LINC00470 is an oncogenic lncRNA in GC, with evidently upregulated expression in GC tissues and cell lines (Yan et al., 2020). It has been found that LINC00470 levels are significantly associated with metastasis, advanced TNM stages, and cancer progression of GC patients. LINC00470 could enhance proliferation, migration, and invasion of GC cells. Mechanistically, LINC0047 recruits the m^6^A writer METTL3 to increases m^6^A modification on *PTEN* mRNA, thereby decreasing its stability. As a result, LINC00470 inhibits its target gene *PTEN* expression and promotes tumorigenesis of GC cells.

### Hepatocellular carcinoma

LncRNA GATA3-AS, the antisense RNA of *GATA3* gene, acts as an oncogene and accelerates metastasis in HCC (Lan et al., 2019b). Indeed, lncRNA GATA3-AS enhances the interaction of KIAA1429, a component of the m^6^A methyltransferase complex, with *GATA3* pre-mRNA as the molecule scaffold. As a result, KIAA1429 induces *GATA3* pre-mRNA methylation on the 3′-UTR, leading the isolation of RNA-binding protein HuR and degradation of *GATA3* pre-mRNA in HCC cells.

### Colorectal cancer

LINRIS is upregulated in human CRC samples compared with their matched adjacent normal tissues. LncRNA LINRIS promotes disease progression by accelerating glycolysis of CRC cells (Wang et al., 2019). LINRIS inhibits K139 ubiquitination of IGF2BP2 protein and prevents the degradation of IGF2BP2 via the autophagy-lysosome pathway. IGF2BP2 can recognize m^6^A-modified mRNAs, such as *ELAVL1*, *HUR*, *MATR3*, and *PABPC1*, and maintain their stability. Interestingly, knockdown of LINRIS lessens the MYC-mediated glycolysis, which is regulated by IGF2BP2 in CRC cells. In summary, LINCRIS regulates the ubiquitination of the crucial m^6^A reader protein IGF2BP2 and the LINRIS-IGF2BP2-MYC axis might be a promising therapeutic target of CRC.

In CRC, LINC021 is remarkably upregulated in CRC tissues compared to normal tissues (Wu et al., 2022). Levels of LINC021 might be an independent prognostic factor of CRC patients. LINC021 functions as an oncogene to promote CRC malignant proliferation, colony formation, migration capabilities, and reduced cell apoptosis. Specifically, oncogenic LINC021 specifically interacts with IGF2BP2 to enhance the mRNA stability of *MSX1* and *JARID2*. These findings demonstrate the important roles of LINC021/IGF2BP2/MSX1 axis in CRC tumorigenesis and shed light for CRC treatment.

## Impacts of m^6^A modifications on miRNAs

**FIGURE 2:**
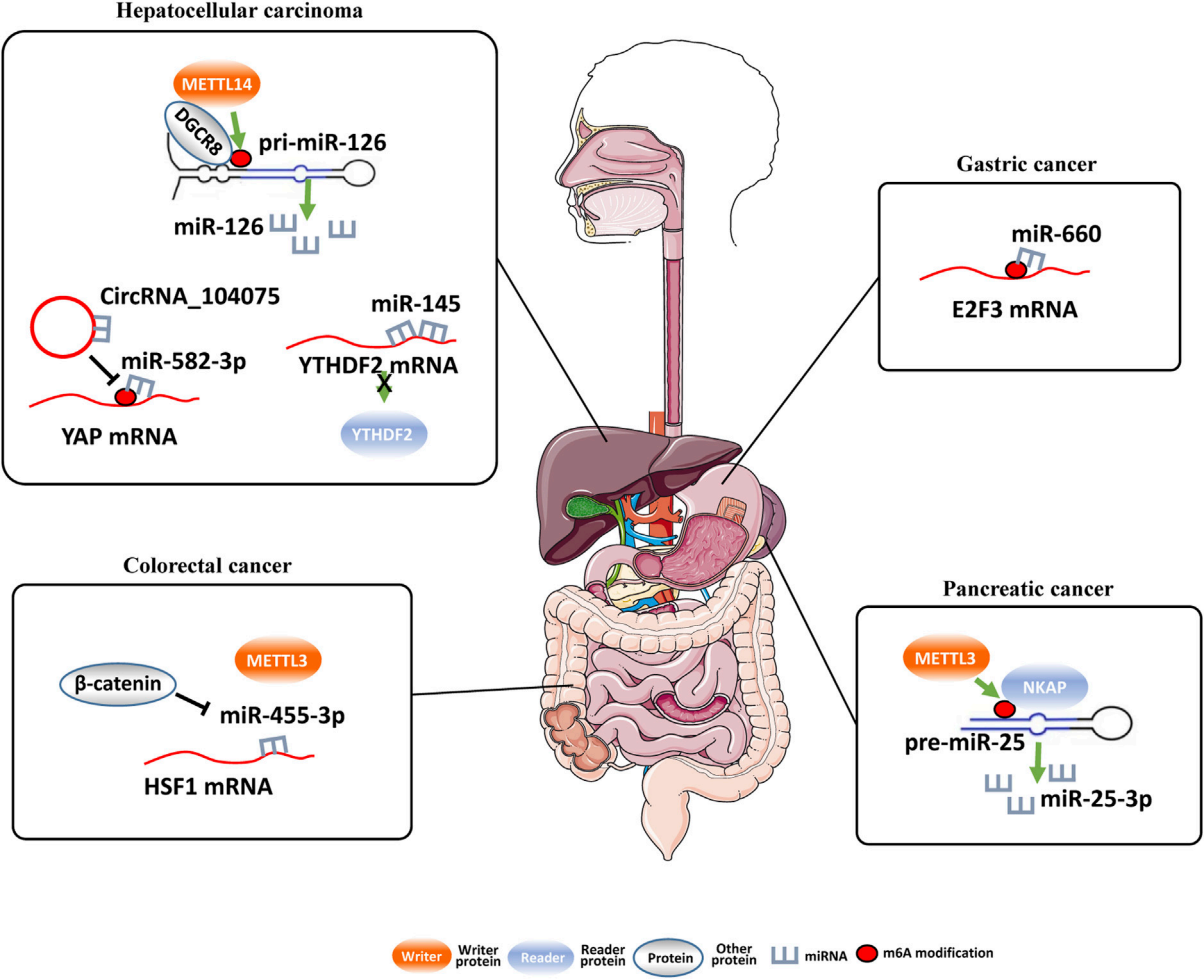
Regulation of miRNAs by m^6^A modification and miRNAs regulate m^6^A modification..

MiRNAs are crucial regulatory small ncRNAs involved in tumorigenesis. M^6^A modification may impact synthesis and functions of miRNAs at various levels (Table 3; Figure 2). On one hand, m^6^A-related proteins regulate the expression of key microprocessor proteins in miRNA biosynthesis, such as Drosha and DGCR8, to affect miRNA expression. On the other hand, m^6^A-related proteins are also target genes of miRNAs in cancers (Burke et al., 2014; Alarcon et al., 2015; Catalanotto et al., 2016).

**TABLE 3:** M^6^A modification regulates miRNA.

Proteins	Protein types	Functions	Target miRNAs	Cancers	Functional classification	References
Unknown	Unknown	M^6^A was necessary for the interaction between miR-660 and E2F3 3′UTR	miR-660	GC	Promoting GC progression	He and Shu (2019)
METTL14	Writer	Promoting the processing of DROSHA into precursor miRNA (pre-miRNA)	miR-126	HCC	Inhibiting the repressive effect of METTL14 in cancer metastasis	Ma et al. (2017)
Unknown	Unknown	M^6^A was necessary for the interaction between miR-582-3 and YAP 3′UTR	miR-582-3p	HCC	Stimulating YAP-dependent tumorigenesis	Zhang et al. (2018)
NKAP	Reader	Promoting miRNA process and maturation of miR-25-3p	miR-25-3p	PC	Promoting transformation process induced by smoking	Zhang et al. (2019a)

GC, gastric cancer; HCC, hepatocellular carcinoma; PC, pancreatic cancer.

### Gastric cancer

MiR-660 is significantly downregulated in GC tissues and associated with advanced TNM stage, larger tumor size, lymph node metastasis, and poor prognosis of GC patients (He and Shu, 2019). In line with these, miR-660 significantly repressed proliferation of GC cells and induced apoptosis of GC cells. In GC cells, miR-660 reduces expression of *E2F3* via directly binding to *E2F3* 3′- UTR. It is worth noting that the m^6^A motif was found in *E2F3* 3′-UTR and the m^6^A site is necessary for the interaction between miR-660 and *E2F3* mRNA. These findings further elucidate the crucial role of m^6^A in GC and highlights the regulatory functions of the miR-660/E2F3 pathway during GC progression.

### Hepatocellular carcinoma

The m^6^A modification is reduced in HCC and METTL14 is the main factor involved in abnormal m^6^A modification (Scholler et al., 2018). As miRNAs play important roles in tumorigenesis and metastasis, Ma et al. (2017) hypothesized that METTL14 might impact HCC progression by regulating miRNAs in an m^6^A-dependent, pri-miRNA-processing manner. Subsequent immunoprecipitation assays demonstrated that METTL14 directly interacted with the microprocessor protein DGCR8. Moreover, the interaction between METTL14 and DGCR8 was suppressed after ribonuclease treatment, elucidating that there were RNAs participating in their interaction. Interestingly, mature miR126 and METTL14 had the similarity expression trend in HCC cells. Consistently, unprocessed pri-miR126 accumulated upon METTL14-depleted cells and accelerated in METTL14-overexpressed cells. The overexpression of METTL14 increased the binding between pri-miR126 and DGCR8. These findings suggest that METTL14 regulated the recognition and binding of DGCR8 to pri-miRNAs to impact pri-miRNA processing in HCC.

Oncogenic circ_104,075 was evidently upregulated in HCC tissues, serum, and cell lines (Zhang et al., 2018). In HCC cells, circ_104,075 absorbed miR-582-3p as a molecular sponge to increase *YAP* expression. There is an m^6^A motif in the 353-357 nt region of *YAP* 3′-UTR, which is essential for the interaction between miR-582-3p and *YAP* 3′-UTR. Interestingly, circRNA_104,075 could be used for prediction of the occurrence of HCC, with the AUC-ROC of 0.973.

### Pancreatic cancer

Cigarette smoke condensate enhanced m^6^A modification of oncogenic miR-25 to promote its maturation in pancreatic duct epithelial cells (Zhang et al., 2019a). MiR-25-3p are upregulated in smokers and in PC tissues, which are significantly assciated with poor prognosis of PC patients. NKAP (NF-κB related protein) is a novel m^6^A reader and recognizes the m^6^A-modified miR-25-3p precursor RNA in pancreatic cancer, leading to accelerated miRNA process and maturation of miR-25-3p and increased expression of miR-25-3p. miR-25-3p suppresses the expression of PHLPP2 and regulates the AKT-p70S6K signaling pathway. These results indicate that important roles of METTL3/miR-25-3p/PHLPP2/AKT axis during the transformation process induced by smoking in PC patients.

## MiRNAs regulate m^6^A modifications

The m^6^A modification-related proteins play important roles in various cancers. Interestingly, dysregulated miRNAs can also regulate expression of these m^6^A modification-related genes and the m^6^A levels in cells, which may lead to tumorigenesis (Cai et al., 2018) (Table 4; Figure 2).

**TABLE 4:** MiRNA regulates m^6^A modification.

miRNAs	Cancers	Proteins	Functions	Functional classification	References
miR-145	HCC	YTHDF2	Decreasing YTHDF2 by targeting 3′UTR of YTHDF2 mRNA	Regulating the level of m^6^A and inhibiting HCC cell proliferation	Yang et al. (2017)
miR-455-3p	CRC	HSF1	Interacting with 3′-UTR of HSF1 mRNA	Interrupting the interaction between METTL3 and HSF1 mRNA	Song et al. (2020)

HCC, hepatocellular carcinoma; CRC, colorectal cancer.

### Hepatocellular carcinoma

Yang et al. investigated the regulatory mechanism of *YTHDF2*, which was poorly understood in HCC (Yang et al., 2017). It has been found that miR-145 modifies m^6^A modification through suppressing *YTHDF2* expression in HCC. On the contrary, inhibition of miR-145 upregulates *YTHDF2* expression levels in HCC cells. In line with these data, the expression levels of miR-145 in HCC tissues was negatively correlated with the level of YTHDF2 mRNA. Additionally, inhibition of miR-145 evidently decreased m^6^A levels, which were rescued by silencing of *YTHDF2* expression in cells. In summary, the miR-145/YTHDF2 axis plays a vital role in modulating m^6^A levels of HCC cells.

### Colorectal cancer

In colorectal cancer cells, miR-455-3p can interact with 3′-UTR of *HSF1* mRNA to repress its translation (Song et al., 2020). The inhibition of WNT/β-catenin signaling by pyrvinium or β-catenin knockdown impaired *HSF1* mRNA translation and its m^6^A modification. Importantly, inhibition of miR-455-3p can rescue the reduction of HSF1 m^6^A modification and METTL3 interaction caused by β-catenin depletion. β-catenin suppressed the biogenesis of miR-455-3p, which interrupt the interaction between METTL3 and *HSF1* mRNA, thus promoting *HSF1* m^6^A modification.

## Conclusion

It has been found that m^6^A modification levels of lncRNAs or miRNAs might impact RNA stability. Conversely, lncRNAs and miRNAs are also involved in regulating m^6^A levels of mRNAs in cells. These m^6^A-related lncRNAs and miRNAs functions as novel oncogenes or rumor suppressors in malignant cells (Table 5). However, there are still no studies on genome-wide screening of crucial lncRNAs or miRNAs involved in regulating m^6^A levels of mRNAs in cancers. Moreover, it is still largely unclear if there are differences on mechanisms of regulating m^6^A modification of lncRNAs, miRNAs or mRNAs in different gastrointestinal cancer cells. In summary, there are still considerable studies on unveiling the regulatory relationships between lncRNAs/miRNAs and m^6^A modification. Though, we are certain of targeting m^6^A-related lncRNAs and miRNAs may provide novel options for gastrointestinal cancer treatments.

**TABLE 5:** Function of lncRNA and miRNA m^6^A modification in gastrointestinal cancers.

Cancers	RNA names	Function of m^6^A	References
ESCC	LINC00022	M^6^A modification in LINC00022 enhances its stability and promotes tumor growth of ESCC	Cui et al. (2021)
GC	NEAT1	Demethylation of NEAT1 promotes GC cell proliferation and migration	Zhang et al. (2019b)
	ARHGAP5-AS1	ARHGAP5-AS1 increases m^6^A modification to enhance chemoresistance of GC cells	Zhu et al. (2019)
	LINC00470	LINC00470 increases m^6^A modification to promote GC cell proliferation and migration	Yan et al. (2020)
	miR-660	M^6^A modification is necessary for miR-660 oncogenic function in GC	He and Shu (2019)
HCC	LINC00958	M^6^A modification in LINC00958 enhances its stability and promotes HCC progression	Zuo et al. (2020)
	GATA3-AS	GATA3-AS induces m^6^A modification to promote proliferation and cancerigenicity of HCC	Lan et al. (2019b)
	miR-126	METTL14 promotes miR-126 processing, and miR-126 promotes HCC metastasis	Ma et al. (2017)
	miR-582-3p	M^6^A modification is necessary for miR-582-3p oncogenic function in HCC	Zhang et al. (2018)
	miR-145	MiR-145 decreases m^6^A modification and inhibited HCC cell proliferation	Yang et al. (2017)
PC	DANCR	M^6^A modification in DANCR enhances its stability and promotes PC progression	Hu et al. (2020)
	DICER1-AS1	M^6^A modification in DICER1-AS1 promotes its degradation to promotes PC progression	Hu et al. (2022)
	KCNK15-AS1	M^6^A modification in KCNK15-AS1 increases its expression and inhibits proliferation and malignant behaviors of PC cells	He et al. (2018), He et al. (2021)
	miR-25-3p	NKAP promotes miR-25-3p processing, and miR-25-3p promotes PC metastasis	Zhang et al. (2019a)
CRC	XIST	M^6^A modification in XIST promotes its degradation to repress CRC progression	Yang et al. (2020)
	GAS5	M^6^A modification in GAS5 promotes its degradation to promotes CRC progression	Ni et al. (2019)
	LINRIS	LINRIS enhances m^6^A- mediated RNA stability to promote aerobic glycolysis in CRC	Wang et al. (2019)
	LINC021	LINC021enhances m^6^A- mediated RNA stability to promote CRC progression	Wu et al. (2022)
	miR-455-3p	MiR-455-3p inhibits HSF1 m^6^A modification to suppress CRC tumor growth	Song et al. (2020)

ESCC, esophageal squamous cell carcinoma; GC, gastric cancer; HCC, hepatocellular carcinoma; PC, pancreatic cancer; CRC, colorectal cancer.

## Abbreviations

m6A, N6-Methyladenosine; lncRNAs, long non-coding RNAs; miRNAs, microRNAs; ncRNAs, non-coding RNAs; ceRNA, competing endogenous RNA; ESCC, esophageal squamous cell carcinoma: GC, gastric cancer; HCC, hepatocellular carcinoma; HDGF, hepatoma-derived growth factor; PC, pancreatic cancer; CRC, colorectal cancer.
