# Supplementary material for: New insights on the interplays between m6A modifications and microRNA or lncRNA in gastrointestinal cancers
Source: Front Cell Dev Biol. 2023 Jun 19;11:1157797. doi: 10.3389/fcell.2023.1157797 (PMC10316788; doi:10.3389/fcell.2023.1157797)
Supplement: Supplementary file 1 [file Table1.DOCX]

**Table 1. M^6^A modification regulates lncRNA stability**

| Proteins | Protein types | Functions | Target lncRNAs | Tissues | Functional classification | References | |
| --- | --- | --- | --- | --- | --- | --- | --- |
| FTO | Eraser | Decreased m^6^A levels | LINC00022 | ESCC | Promoting tumor growth of ESCC | ([Cui et al., 2021](#_ENREF_6)) |  |
| ALKBH5 | Eraser | Decreased m^6^A levels | NEAT1 | GC | Promoting GC cell proliferation and migration | ([Zhang et al., 2019b](#_ENREF_42)) |  |
| METTL3 | Writer | Increased m^6^A levels | LINC00958 | HCC | Promoting HCC cell proliferation and migration | ([Zuo et al., 2020](#_ENREF_46)) |  |
| IGF2BP | Reader | Identify the m^6^A site | DANCR | PC | Promoting cell proliferation and stem cell-like properties | ([Hu et al., 2020](#_ENREF_13)) |  |
| YTHDF3 | Reader | Identify the m^6^A site | DICER1-AS1 | PC | Suppressing glycolysis, proliferation, and metastasis of PC cells | ([Hu et al., 2022](#_ENREF_14)) |  |
| ALKBH5 | Eraser | Decreased m^6^A levels | KCNK15-AS1 | PC | Acting as a tumor suppressor and inhibits malignant behaviors of PC cells | ([He et al., 2018](#_ENREF_11))  ([He et al., 2021](#_ENREF_12)) |  |
| METTL14 | Writer | Increased m^6^A levels | XIST | CRC | Promoting CRC cell proliferation and migration | ([Yang et al., 2020](#_ENREF_38)) |  |
| YTHDF3 | Reader | Identify the m^6^A site | GAS5 | CRC | Inhibiting cell proliferation, invasion, migration, EMT, and radiation resistance | ([Ni et al., 2019](#_ENREF_24)) |  |

ESCC, Esophageal squamous cell carcinoma; GC, Gastric cancer; HCC, Hepatocellular carcinoma; PC, Pancreatic cancer; CRC, Colorectal cancer

**Table 2. LncRNA-regulated m^6^A modification**

| LncRNAs | Cancers | | Proteins | Functions of lncRNAs | Functional classification | References |
| --- | --- | --- | --- | --- | --- | --- |
| ARHGAP5-AS1 | GC | METTL3  HUR | | Recruiting METTLE3 and stimulating m^6^A modification on ARHGAP5 mRNA | Enhancing chemoresistance of GC cells | ([Zhu et al., 2019](#_ENREF_45)) |
| LINC00470 | GC | METTL3  YTHDF2 | | Increasing m^6^A modification on PETN mRNA decreasing its stability and degrading m^6^A-dependent reader protein YTHDF2. | Promoting GC cell proliferation and migration | ([Yan et al., 2020](#_ENREF_37)) |
| GATA3-AS | HCC | KIAA1429 | | Acting as a cis-acting element for the interaction of KIAA1429 with GATA3 pre-mRNA. | Promoting the proliferation and cancerigenicity of HCC | ([Lan et al., 2019b](#_ENREF_20)) |
| LINRIS | CRC | IGF2BP2 | | Reducing IGF2BP2 ubiquitination | Promoting aerobic glycolysis in CRC | ([Wang et al., 2019](#_ENREF_32)) |
| LINC021 | CRC | IGF2BP2 | | Binding with the m^6^A reader IGF2BP2 protein and enhanced the mRNA stability of MSX1 and JARID2 | Promoting CRC malignant proliferation, migration capabilities, and reduced cell apoptosis. | ([Wu et al., 2022](#_ENREF_35)) |

GC, Gastric cancer; HCC, Hepatocellular carcinoma; CRC, Colorectal cancer

**Table 3. M^6^A modification regulates miRNA**

| Proteins | Protein types | Functions | Target miRNAs | Cancers | Functional classification | References |
| --- | --- | --- | --- | --- | --- | --- |
| Unknown | Unknown | M^6^A was necessary for the interaction between miR-660 and E2F3 3’UTR | miR-660 | GC | Promoting GC progression | ([He and Shu, 2019](#_ENREF_10)) |
| METTL14 | Writer | Promoting the processing of DROSHA into precursor miRNA (pre-miRNA). | miR-126 | HCC | Inhibiting the repressive effect of METTL14 in cancer metastasis | ([Ma et al., 2017](#_ENREF_21)) |
| Unknown | Unknown | M^6^A was necessary for the interaction between miR-582-3 and YAP 3’UTR | miR-582-3p | HCC | Stimulating YAP-dependent tumorigenesis | ([Zhang et al., 2018](#_ENREF_44)) |
| NKAP | Reader | Promoting miRNA process and maturation of miR-25-3p | miR-25-3p | PC | Promoting transformation process induced by smoking | ([Zhang et al., 2019a](#_ENREF_41)) |

GC, Gastric cancer; HCC, Hepatocellular carcinoma; PC, Pancreatic cancer

**Table 4. MiRNA regulates m^6^A modification**

| miRNAs | Cancers | Proteins | Functions | Functional classification | References |
| --- | --- | --- | --- | --- | --- |
| miR-145 | HCC | YTHDF2 | Decreasing YTHDF2 by targeting 3’UTR of YTHDF2 mRNA | Regulating the level of m^6^A and inhibiting HCC cell proliferation | ([Yang et al., 2017](#_ENREF_39)) |
| miR-455-3p | CRC | HSF1 | Interacting with 3’-UTR of HSF1 mRNA | Interrupting the interaction between METTL3 and HSF1 mRNA | ([Song et al., 2020](#_ENREF_30)) |

HCC, Hepatocellular carcinoma; CRC, Colorectal cancer

**Table 5. Function of lncRNA and miRNA m^6^A modification in gastrointestinal cancers**

| Cancers | RNA names | Function of m^6^A | References |
| --- | --- | --- | --- |
| ESCC | LINC00022 | M^6^A modification in LINC00022 enhances its stability and promotes tumor growth of ESCC | ([Cui et al., 2021](#_ENREF_6)) |
| GC | NEAT1  ARHGAP5-AS1  LINC00470  miR-660 | Demethylation of NEAT1 promotes GC cell proliferation and migration  ARHGAP5-AS1 increases m^6^A modification to enhance chemoresistance of GC cells  LINC00470 increases m^6^A modification to promote GC cell proliferation and migration  M^6^A modification is necessary for miR-660 oncogenic function in GC | ([Zhang et al., 2019b](#_ENREF_42))  ([Zhu et al., 2019](#_ENREF_45))  ([Yan et al., 2020](#_ENREF_37))  ([He and Shu, 2019](#_ENREF_10)) |
| HCC | LINC00958  GATA3-AS  miR-126  miR-582-3p  miR-145 | M^6^A modification in LINC00958 enhances its stability and promotes HCC progression  GATA3-AS induces m^6^A modification to promote proliferation and cancerigenicity of HCC  METTL14 promotes miR-126 processing, and miR-126 promotes HCC metastasis  M^6^A modification is necessary for miR-582-3p oncogenic function in HCC  MiR-145 decreases m^6^A modification and inhibited HCC cell proliferation | ([Zuo et al., 2020](#_ENREF_46))  ([Lan et al., 2019b](#_ENREF_20))  ([Ma et al., 2017](#_ENREF_21))  ([Zhang et al., 2018](#_ENREF_44))  ([Yang et al., 2017](#_ENREF_39)) |
| PC | DANCR  DICER1-AS1  KCNK15-AS1  miR-25-3p | M^6^A modification in DANCR enhances its stability and promotes PC progression  M^6^A modification in DICER1-AS1 promotes its degradation to promotes PC progression  M^6^A modification in KCNK15-AS1 increases its expression and inhibits proliferation and malignant behaviors of PC cells  NKAP promotes miR-25-3p processing, and miR-25-3p promotes PC metastasis | ([Hu et al., 2020](#_ENREF_13))  ([Hu et al., 2022](#_ENREF_14))  ([He et al., 2018](#_ENREF_11))  ([He et al., 2021](#_ENREF_12))  ([Zhang et al., 2019a](#_ENREF_41)) |
| CRC | XIST  GAS5  LINRIS  LINC021  miR-455-3p | M^6^A modification in XIST promotes its degradation to repress CRC progression  M^6^A modification in GAS5 promotes its degradation to promotes CRC progression  LINRIS enhances m^6^A- mediated RNA stability to promote aerobic glycolysis in CRC  LINC021enhances m^6^A- mediated RNA stability to promote CRC progression  MiR-455-3p inhibits HSF1 m^6^A modification to suppress CRC tumor growth | ([Yang et al., 2020](#_ENREF_38))  ([Ni et al., 2019](#_ENREF_24))  ([Wang et al., 2019](#_ENREF_32))  ([Wu et al., 2022](#_ENREF_35))  ([Song et al., 2020](#_ENREF_30)) |

ESCC, Esophageal squamous cell carcinoma; GC, Gastric cancer; HCC, Hepatocellular carcinoma; PC, Pancreatic cancer; CRC, Colorectal cancer
